# Assessing key cost drivers associated with caring for chronic kidney disease patients

**DOI:** 10.1186/s12913-016-1922-4

**Published:** 2016-12-28

**Authors:** Paul Damien, Holly J. Lanham, Murali Parthasarathy, Nikhil L. Shah

**Affiliations:** 1 McCombs School of Business, University of Texas in Austin, Austin, USA; 2 The University of Texas Health Science Center San Antonio, San Antonio, USA; 3 South Texas Veterans Health Care System, San Antonio, USA; 4 Saaraa Medical Solutions, Whitehouse Station, NJ USA; 5 Piedmont Clinic, Atlanta, GA USA; 6 Georgia Institute of Technology, Atlanta, GA USA

**Keywords:** Chronic kidney disease, Total costs drivers, Co-morbid conditions

## Abstract

**Background:**

To examine key factors influencing chronic kidney disease (CKD) patients’ total expenditure and offer recommendations on how to reduce total cost of CKD care without compromising quality.

**Methods:**

Using the 2002–2011 Medical Expenditure Panel Survey (MEPS) data, our cross-sectional study analyzed 197 patient records—79 patients with one record and 59 with two entries per patient (138 unique patients). We used three patient groups, based on international statistical classification of diseases version 9 code for condition (ICD9CODX) classification, to focus inference from the analysis: (a) non-dialysis dependent CKD, (b) dialysis and (c) transplant. Covariate information included region, demographic, co-morbid conditions and types of services. We used descriptive methods and multivariate generalized linear models to understand the impact of cost drivers. We compared actual and predicted CKD cost of care data using a hold-out sample of nine, randomly selected patients to validate the models.

**Results:**

Total costs were significantly affected by treatment type, with dialysis being significantly higher than non-dialysis and transplant groups. Costs were highest in the West region of the U.S. Average costs for patients with public insurance were significantly higher than patients with private insurance (*p* < .0743), and likewise, for patients with co-morbid conditions over those without co-morbid conditions (*p* < .001).

**Conclusions:**

Managing CKD patients both before and after the onset of dialysis treatment and managing co-morbid conditions in individuals with CKD are potential sources of substantial cost savings in the care of CKD patients. Comparing total costs pre and post the United States Affordable Care Act could provide invaluable insights into managing the cost-quality tradeoff in CKD care.

## Background

Chronic kidney disease (CKD) affects approximately 10% of the population worldwide, and millions die each year because they do not have access to affordable treatment [1]. The global costs of CKD quadrupled in the 20 years leading up to 2001 [2] and are expected to continue to increase due to world population growth and aging [3–5]. According the 2010 Global Burden of Disease study, CKD was ranked 27^th^ in the list of causes of total number of deaths worldwide in 1990, and rose to 18^th^ in 2010 [6]. Over 80% of patients who receive treatment for kidney failure live in affluent countries with universal access to health care and large elderly populations [6]. Patients with CKD incur 85% higher costs and 50% higher government subsidies than those without CKD, and costs of care increase by CKD stage [7]. In the United States, treatment of CKD is estimated at $48 billion per year, consuming 6.7% of the total Medicare budget to care for less than 1% of the covered population; and China’s economy is estimated to lose $558 billion over the next decade due to morbidity and mortality attributable to heart disease and kidney disease [1]. Understanding the key cost drivers associated with caring for individuals with CKD could suggest new strategies to bend the cost curve of this complex and debilitating condition.

The severity of CKD is classified into five stages with stage 1 constituting the mildest disease state where few clear symptoms are present, and stage 5 constituting severe illness with poor life expectancy if left untreated. Individuals with stage 5 CKD typically require one of two types of renal replacement therapy, namely dialysis or transplant. These individuals are further classified as having End-Stage Renal Disease (ESRD). Individuals diagnosed with CKD stages 1 to 4 and do not require life-supporting treatments for renal failure are labeled Non-Dialysis Dependent CKD (NDD-CKD). At times, even stage 5 patients that have not yet started renal replacement therapy are grouped under NDD-CKD.

Managing the tradeoff between costs and quality in health care in general is a tremendous challenge [8–12]. Managing the cost/quality tradeoff in patients with CKD presents a unique set of challenges [13, 14], including an increased burden of CKD patients in the world population and the rising expenses associated with it. In 2011, the Fresenius Medical Care worldwide network, estimated that roughly 2.8 million patients globally were being treated for ESRD with a 6–7% growth annually. Of these, approximately 2.1 million were undergoing hemodialysis or peritoneal dialysis, and around 622,000 were living with kidney transplants. The prevalence of treated ESRD patients in the general population shows high global variation, ranging from under 100 to over 2,000 patients per million populations (pmp). ESRD prevalence is highest in Taiwan with around 2,850 pmp, closely followed by Japan with around 2,490 pmp and the US with around 1,970 pmp. Of the CKD cases in the US, about 65% were identified as undergoing dialysis and 30% were identified as living with kidney transplants.

In 2011, Medicare spending in the U.S. per patient, per year, by renal replacement therapy type was: hemodialysis - $87,945; peritoneal dialysis - $71,630; and transplant - $32,922. To curb rapid spending growth in dialysis costs in the Medicare patient population, CMS implemented prospective bundled payments and pay-for-performance incentives. It is unclear whether these strategies will reduce the total cost of dialysis or their effect on quality of care [15]. Additional research is needed to better understand the costs associated with caring for individuals with CKD [16].

A key step in devising cost-efficient strategies for providing high quality care for patients with CKD is identifying the major drivers of cost and their attendant implications for cost of services for each patient. The primary objective of this study is to evaluate the total health care cost for patients with CKD by identifying the drivers of total cost for these patients. Once such factors are determined, the study quantifies, where possible, the reduction in costs per CKD patient associated with these factors. Such inferences could help policy, practice and research stakeholders develop more effective protocols to better manage the cost of caring for patients with CKD without compromising quality. To better focus the inferences drawn from these analyses, we consider three patient groups based on the above classifications: (a) NDD-CKD; (b) Dialysis; and (c) Transplant patients. These are the most commonly defined and used patient groups in the context of worldwide treatment of CKD patients, and hence they form the basis for this research.

## Methods

### Study design

This was a cross-sectional study of CKD adults using a nationally representative Medical Expenditure Panel Survey (MEPS) from 2002 to 2011. MEPS is co-sponsored by the Agency of Healthcare Research and Quality (AHRQ) and the National Center for Health Statistics. The MEPS survey, initiated in 1996, has collected data annually that can be utilized to provide nationally representative estimates of the intensity, frequency, and the cost of healthcare services that Americans use and how these services are covered and paid for by different insurance providers. MEPS data can be accessed through the website administered by AHRQ at www.meps.ahrq.gov. Data from children were not included in our analysis.

### Study data

We started out with an analysis of 211 patients whose data were collected over a 10-year period and ensured there were sufficient number of patients in each year, and that no one year represented less than 6% or more than 13% of the sample. Data were also balanced for the region of the country, gender, marital status, ethnicity, education, patient health status, hospital visits and costs. Duplicate records were then deleted bringing down the number of records to 197. Thus, after carefully eliminating a multitude of recording errors, there were 197 patient records—79 patients with one record and 59 with two entries per patient for a total of 138 patients. To be sure, what we have is a patient level analysis, but since some patients have multiple cost records, we have a repeated measures dataset; hence, at times, we use the phrase ‘patient record’. These patients were segmented into Dialysis, Transplant and Non-Dialysis Dependent CKD (NDD-CKD) treatment groups based on ICD9CODX classification – International Classification of Disease, 9th Revision. Recall that those undergoing the former two treatment types are also described as ESRD patients.

The response or dependent variable of interest in this study is the *Total Expenditure* (or *Total Costs*) incurred by each CKD patient each year, which is the sum of the following expenses: Emergency, Inpatient, Outpatient, Office visits, Medical equipment/supply, Prescription drug, and Other home healthcare. In the time frame considered, it should be noted that almost half of the patients had only one record of Total Costs while the remainder had two. The database did not contain any information as to when patients died.

Typically, health care costs (both charges and payments) are collected for all persons for each medical event they experience in the year, including the amount from each payment source. Charges are the dollar amounts asked ("charge") for a service by a health care provider. This is often different from the actual payments made to providers. Expenditure estimates are based on payments, not charges. More specifically, expenditures in MEPS are comprised of direct payments for care provided during the year, including out-of-pocket payments and payments by private insurance, Medicaid, Medicare, and other sources. All payments were inflation adjusted to target year using the annual PPI Index from the Bureau of Labor Statistics. Bringing in normative external data introduces additional variation which we sought to avoid.

MEPS also includes covariate information on household income, medical conditions and clinical classification codes. Data on demographic and socioeconomic variables, such as gender, age, race, family income, region, insurance coverage are available for respondents and families residing in the U.S. To understand how to better minimize total costs, it is important to examine the influence of covariates on costs. To this end, based on targeted discussions with health care providers and CKD specialists, we categorized these covariates into five major *groups*.

1. REGION: Northeast, South, Midwest, and West in the United States
2. TREATMENT TYPE: Dialysis, Transplant, and NDD-CKD
3. DEMOGRAPHIC data on each patient: gender, race, age, family income, source of care provider, and health insurance coverage. Gender, age, and health insurance coverage feature only in the descriptive analysis, since they did not correlate to expenditure. Furthermore, since the number of non-whites and non-blacks in the final sample were very few, without loss of generality, we grouped ‘whites’ and ‘other non-blacks’ into the reference group. From a multivariate analysis perspective, the inferences and conclusions would be identical if we had grouped ‘blacks’ and ‘other non-whites’ as the reference group. The only changes would be in the magnitudes and signs of the intercepts and slope parameters.
4. CO-MORBID conditions for each patient: diabetes, high blood pressure, other heart disease
5. TYPE OF SERVICES used by a patient: Costs associated with office-based visits; outpatient visits; hospital discharges; nights in hospitals; and prescription medications were the most predominant in the list of costs for each patient, and hence these factored into the multivariate analysis.

Details on certain key variables from the above list used in the study are provided below:

(A) Insurance Coverage: Individuals were classified into the following three insurance categories based on household responses to the health insurance status questions.
*Any private health insurance:* Individuals who, at any time during the year, had insurance that provided coverage for hospital and physician care (other than Medicare, Medicaid, or other public hospital/physician coverage) were classified as having private insurance. Coverage by TRICARE (Armed Forces-related coverage) was also included as private health insurance. Insurance that provided coverage for a single service only, such as dental or vision coverage, was not included.
*Public coverage only:* Individuals were considered to have public coverage only if they met both of the following criteria: 1) they were not covered by private insurance at any time during the year, and 2) they were covered by one of the following public programs at some point during the year: Medicare, Medicaid, or other public hospital/physician coverage.
*Uninsured:* Individuals were considered uninsured if they were not covered by private hospital/physician insurance, Medicare, TRICARE, Medicaid, or other public hospital/physician programs at any time during the entire year or their period of eligibility for the survey. There were only two uninsured patients in this study. Without loss of generality, therefore, we pooled them with those in the public insurance group.
(B) Office-based visit costs included expenses for visits to both physician and non-physician medical providers seen in office settings.
*Hospital inpatients costs* included room and board and all hospital diagnostic and laboratory expenses associated with the basic facility charge, payments for separately billed physician inpatient services, and some emergency care expenses incurred immediately prior to inpatient stays.
*Hospital outpatient costs* included expenses for visits to both physicians and other medical providers seen in hospital outpatient departments, including payments for services covered under the basic facility charge and those for separately billed physician services.
*Emergency care costs* included payments for services covered under the basic facility charge and those for separately billed physician services, but excluded expenses for emergency room services that were included in a hospital inpatient admission.
*Prescribed medicines costs* included expenses for all prescribed medications that were initially purchased or refilled during the year, as well as expenses for diabetic supplies.
*Ambulatory costs* combined office-based, hospital outpatient, and emergency room expense categories described above.
*Home health costs* include expenses for home care provided by agencies and independent providers.
*Other costs* include expenses for care in all categories not specified as a separate category including those for miscellaneous medical equipment and supplies.

To complete the data discussion, we now focus on the *type of variables* (categorical vs. continuous) under Groups 2 through 5 above to be used in the multivariate analysis. Many of the variables under Groups 2 through 5 are categorical. To include these in the multivariate analysis, dummy (or 0–1 binary) variables were created.

*Group 2 categorical variables*: NDD-CKD was used as the reference group to be used as the basis of comparison with Dialysis and Transplant patients.

*Group 3 categorical variables*: Non-black was the reference group for race. Low income patients (annual family income of less than $45000) were treated as the reference group versus high income patients.

*Group 4 variables*: For all the covariates in this collection, the reference groups, coded 0, corresponded to absence of the clinical conditions—diabetes, high blood pressure and other heart conditions.

*Group 5 variables*: None were categorical.

In the multivariate analyses, Models 1 through 4 correspond, respectively, to the covariate analyses resulting from the variables detailed above under Groups 2 through 5.

### Statistical analyses

**Fig. 1:**
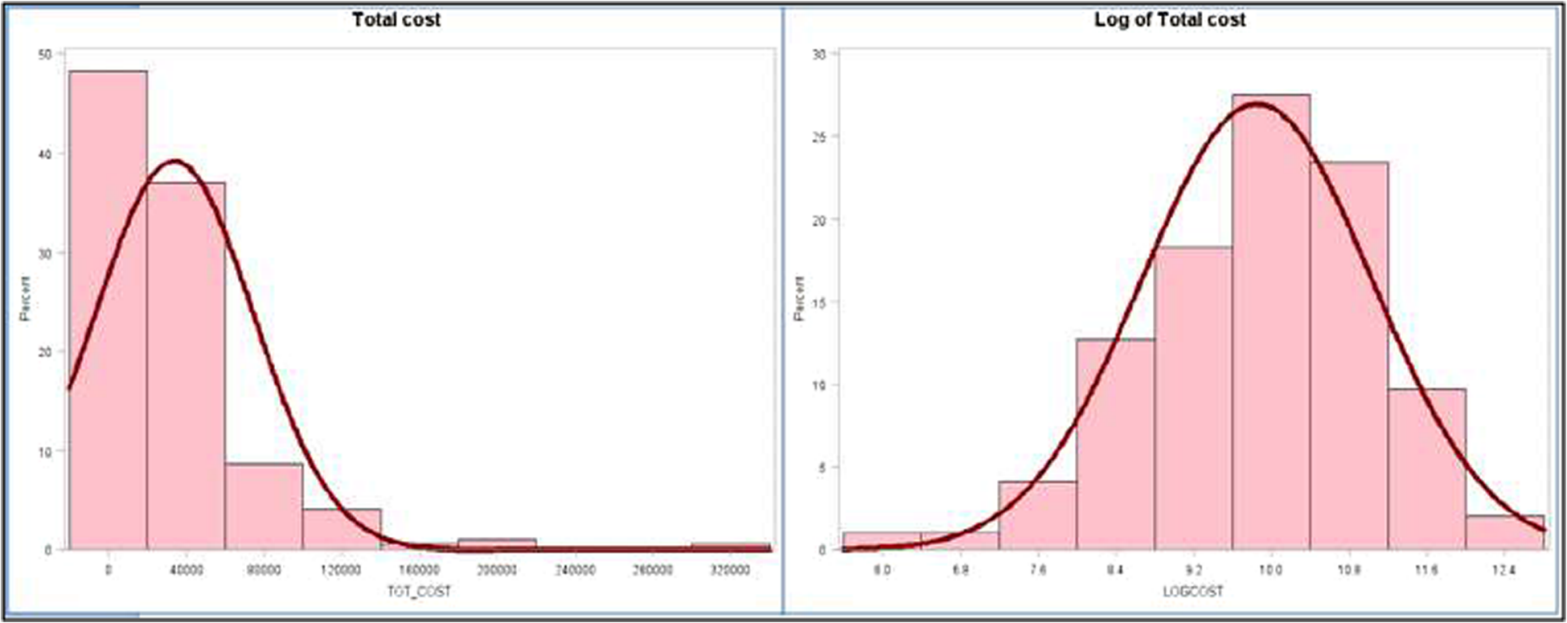
Histogram of total patient cost before and after logarithmic transformation

Consider Panel a in Fig. 1 that depicts the unimodal histogram of the Total Cost data used in this study where these data range from $294 to $335,000. Since a few patients tend to have much higher costs than the rest, the histogram is skewed right. This suggests that a natural log transformation of the data would be appropriate to ensure approximate normality [17]. Panel b in Fig. 1 shows the Total Cost data post log transformation. Throughout, we work with these log-transformed Total Cost data as the dependent variable (**Y**) in the four log-linear models described later. In these models, Region and Health Insurance Coverage were omitted since they were best addressed by descriptive analysis, as shown later in the paper. Additionally, we found that, as covariates, these two variables do not correlate with total expenditure data.

#### Model validation

The multivariate log-linear models are further used to predict or forecast the total expenditures for a hold-out sample of nine, randomly selected patients. In addition to illustrating the feasibility and robustness of the methodology, such ex ante inferences could prove useful to patients and providers to better plan ahead.

## Results

The results are classified into descriptive and multivariate where the latter are based on the log-linear statistical models. The descriptive results complement the inferences from the model-based analysis. Where appropriate, pairwise t-tests were carried out to check for statistical significance of the descriptive results and, in the following, these are indicated via *p*-values.

### Descriptive results

Exploratory inferences from the data for each of the five groups detailed earlier are first described since they often provide useful insights, and also serve as preliminary analytic steps to help build multivariate models.

#### Region

Out of 197 patient records, 46% were from South, 23% from West, 22% from Midwest and 9% from Northeast. Total patient cost is found to be highest and statistically significant in the West ($39,905) and least in the South ($30,239) with *p*-value < .05. In percent terms, compared to the West, cost of treatment in the: South is 28% lower; North-East is 20% lower; Midwest is 12% lower. To control for the known Medicare allowable increase in payments to different regional markets and to avoid the subsequent bias in unadjusted dollar figures, all payments were inflation adjusted to target year using annual Cost of Living for the specific region from the Bureau of Labor Statistics.

#### Treatment type

Of the 197 records, 43% corresponded to Dialysis patient visits and 22% were Transplant patient visits. Dialysis patients experienced significantly higher (*p*-value < .001) average expenditures ($48,225) compared to NDD-CKD ($27,862) or Transplant ($16,190) patients.

#### Demographic

Men accounted for 62% while women accounted for 38% of the costs. In terms of race, Black patients topped the list with 41% of costs. 98% of costs were incurred by patients that have source of care providers while 2% had no providers. Finally, patients with public insurance had significantly higher costs than those with private insurance (*p* < .0743). The key reason for this result stems from the fact that the cost of office-based visits for the former group of patients was, on the average, $8239 more than the latter group. Other researchers have seen mixed results in this regard since insurance related conclusions could vary from country to country.

#### Co-morbid conditions

Hypertension, Diabetes and Heart-Related conditions were the most common co-morbid conditions associated with CKD patients. About 90% have high BP, 44% suffer from Diabetes and 61% have heart related disease. Average expenditure for patients with co-morbid conditions ($95,749) was significantly higher (*p*-value < .001) than those without co-morbid conditions ($36,360).

#### Types of services

Some conclusions regarding the top three service related expenditures are the following. Rank ordering the average costs for the three treatment groups from highest to lowest, we found:

- Office-based visits, in-patient, and out-patient services contribute most to costs incurred by Dialysis patients.
- For NDD-CKD patients, the rank order is office-based visits, prescription, and outpatient costs.
- For Transplant patients, the top three costs in decreasing order are prescription, office-based visits, and outpatient.

### Multivariate models and results

Recall that “Y” refers to the natural logarithm of Total Costs. Figure 1 depicts the unimodal histogram of the Total Cost data used in this study where these data range from $294 to $335,000. Since a few patients tend to have much higher costs than the rest, the histogram is skewed right. Hence, to enable robust statistical analyses, a natural log transformation of the data to ensure approximate normality is required Fox [12]. In all the analyses discussed in the paper, we worked with these log-transformed Total Cost data as the dependent variable (**Y**) in the four models given below. This means the regression coefficients would have to be expressed as percent changes in the untransformed dependent variable (Total Costs); see Fox [17] for details. Note that collapsing all the four models into one would be statistically unsound since the sample size would have to be increased substantially in order to keep the ratio of number of independent variables to sample size large enough to obtain adequate statistical accuracy and power; see, Fox [17] for a discussion on sample size requirements in regression analysis.

The entire analysis was carried out in SAS.

#### Model 1: Y vs. TREATMENT TYPE

$$ \operatorname{Y} = \beta 0+{\beta}_1\operatorname{Transplant}+{\beta}_2\operatorname{Dialysis} $$

- NDD-CKD patients are used as the reference group for the treatment type categorical variable

#### Model 2: Y versus DEMOGRAPHIC FACTORS

$$ \operatorname{Y} = \beta 0+{\beta}_1\operatorname{Black}+{\beta}_2\operatorname{Low} \operatorname {Income} $$

- Race: Non-Black patients comprise the reference group
- Income: High income patients are the reference group

#### Model 3: Y versus CO-MORBID CONDITIONS

$$ \operatorname{Y} = \beta 0+{\beta}_1\operatorname{Diabetes}+{\beta}_2\operatorname{High} \operatorname {BP}+{\beta}_3\operatorname{Other} \operatorname {Heart} \operatorname {Conditions} $$

- Patients with the condition are compared to patients without these conditions (the reference groups)

#### Model 4: Y versus SERVICE TYPE

$$ \operatorname{Y} = \beta 0+{\beta}_1\operatorname{Office} \operatorname {Visits}+{\beta}_2\operatorname{Outpatient}+{\beta}_3\operatorname{Hospital} \operatorname {Discharge}+{\beta}_4\operatorname{Nights} \operatorname {in} \operatorname {Hospitals}+{\beta}_5\operatorname{Prescription} \operatorname {Meds} $$

#### Model 1: Y versus Treatment Type

Consider Table 1. Compared to NDD-CKD patients we found that the Total Cost of treating transplant patients is 44% lower (*p* < .0267). Also, Total Cost of treating dialysis patients is 53% higher (*p* < .0017) than NDD-CKD patients.

**Table 1:** Parameter estimates for Model 1: Treatment Type; Model 2: Demographic Factors

	Parameter	Estimate	Standard Error	*P*-value
Model 1	Intercept	10.05	0.20	<.0001
	Transplant	−0.44	0.20	0.0267
	Dialysis	0.53	0.17	0.0017
Model 2	Intercept	9.52	0.14	<.0001
	Black	0.47	0.17	0.0062
	Low Income	0.34	0.18	0.0570

Model 1: The reference group is Non-dialysis Dependent- CKD patients (NDD-CKD)

Model 2 Reference Groups: The reference group is non-black. Note that this group comprised primarily of white individuals. Since there were very few records corresponding to other ethnicities in the sample, it was appropriate to collapse them into the reference group. For the income variable, High income is the reference group

#### Model 2: Y versus Demographic Factors

Again, consider Table 1. Cost of treating CKD is 47% higher for black patients than non-black patients (*p* < .0062). Furthermore, compared to high income patients, low income patients’ costs are 34% higher (*p* < .0570).

#### Model 3: Y versus Co-morbid Conditions

Now, consider Table 2. Cost of treating CKD patients with high blood pressure is 44% higher when compared to patients with normal blood pressure. Diabetic CKD patients incur 39% more in costs than non-diabetic CKD patients. A final inference for Co-morbid Conditions is that compared to CKD patients without heart disease, costs for those with heart disease are 49% higher.

**Table 2:** Parameter estimates for Model 3: Co-morbid Conditions; Model 4: Types of Services

	Parameter	Estimate	Standard Error	*P*-value
Model 3	Intercept	9.07	0.41	<.0001
	Diabetes	0.39	0.16	0.0144
	High BP	0.44	0.26	0.0902
	Other heart conditions	0.49	0.16	0.0027
Model 4	Intercept	8.912	0.117	<.0001
	Office based visits	0.007	0.001	<.0001
	Outpatient visits	0.008	0.002	0.0006
	Hospital discharges	0.125	0.056	0.0273
	Nights in hospital	0.008	0.004	0.0751
	# Prescribed Medications including refills	0.004	0.001	0.0026

In Model 3, Diabetes, High BP and Other heart disease patients are compared to patients without these conditions (the reference groups)

#### Model 4: Y versus Types of Services

From Table 2, we have the following marginal effects:

- For every additional in-patient visit in the year, patient expenses rises by 13%
- For every additional outpatient visit in the year, patient expenses rises by 1%
- For every additional office-based visit in the year, patient expenses rises by 1%
- For every additional hospital night stay in the year, patient expenses rises by 0.8%
- For every additional prescription medicine in the year, patient expenses rises by 0.4%

### Out-of-sample forecasting results

Since practitioners and patients would be well-served to know what expenditures are likely to be, it is useful to examine how well the statistical models are able to predict or forecast expenditures for a given patient. To accomplish this task, we set aside the actual costs incurred by nine randomly selected patients of which there were six CKD patients, two Dialysis patients and 1 NDD-CKD patient. Using the estimated regression models given above, we predicted the total costs for each patient under each of the four models, and the corresponding 95% prediction interval for these nine point forecasts.

**Fig. 2:**
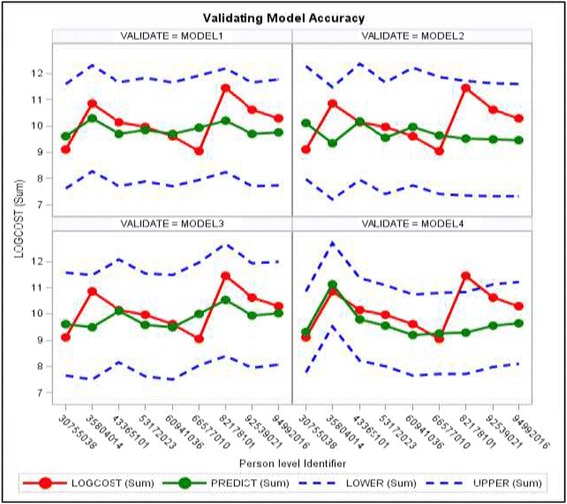
Predicted costs for nine, randomly selected patients

Recalling that the dependent variable is the natural logarithm of Total Costs, consider Fig. 2 below, where the vertical axis is the natural log of Total Costs, and the horizontal axis is the patient ID number. The top left and right panels correspond to the predictions from Model 1 and Model 2, respectively, while the bottom left and right panels correspond to the predictions from Model 3 and Model 4, respectively. In each panel, the outer most curves are the 95% prediction limits for the Total Costs, and the red and green lines correspond to the actual and predicted costs for each of the nine patients, respectively. The models generally tend to underestimate expenditures for three patients, while for the rest the predicted values track the actual expenditures somewhat closely. These patients’ costs were among those extreme expenditure values in the sample that resulted in the histogram of total costs to be skewed right, thereby requiring a natural logarithm transformation of the dependent variable. Overall, the four models perform satisfactorily using a predictive criterion.

## Discussion

Our findings suggest that CKD patient costs are significantly affected by treatment type, namely dialysis, transplant, and non-dialysis dependent CKD; costs differed by region (highest in the West region of the U.S.); and average expenditure for patients with co-morbid conditions is significantly higher than those without co-morbid conditions. Also, patients with public insurance incurred higher costs than those with private insurance, a finding that adds to the mixed results in the literature on cost variation between publically and privately insured individuals [18, 19]. The finding that costs are different by geographic region is consistent with the body of knowledge on geographic variation in care [20–22], and that topic has been well researched. Targeting decision-making units rather than geographic units has been recently discussed in the literature as a way to reduce variation and total cost in health care spending [23]. Because most medical decisions are made by individuals or small groups of individuals rather than by geographic units, findings ways to improve care coordination across medical specialties and use real-time data sharing to support communication and group decision making among multiple providers seems important for improving cost effective care for patients with CKD. The following discussion mainly focuses on the treatment of CKD patients on dialysis and on managing CKD patients with co-morbid conditions because of the relatively higher costs these patients incur.

Given that the total cost of patients on dialysis is significantly higher in comparison to NDD-CKD and transplant patients, CKD specialists and policy and decision-makers should focus attention on managing the expenditures of dialysis patients. It is not surprising that once technology (i.e. a dialysis machine) is introduced in caring for the sickest patients or the most severe of cases, health care costs escalate. In some conditions, where patient costs dramatically escalate, investment in technology and expenditures on invasive procedures for the “sickest of the sick”, it could be argued, does pay off [24]. An example of this is the use of Biventricular heart assist devices (BiVad) in the congestive heart failure population. For patients with the most severe illness, the BiVad offers a decrease in hospitalization rates, but at considerable upfront costs. In contrast, if one considers CKD and dialysis, there has only been a small incremental improvement in the mortality rate of dialysis patients. The prevalence of End Stage Renal Disease is about seven percent per annum and the longevity of the average dialysis patient is five years. These numbers are actually quite interesting, if one considers the CKD/dialysis mortality rate that amounts to a 20% drop off each year, which is worse than most forms of cancer and many other chronic diseases.

The prevalence of stage 5 CKD is increasing and costs are escalating with the steepest portion of the costs for a dialysis patient being incurred during the first 180 days of dialysis treatment. This suggests that one potentially impactful strategy for decreasing these costs is to ensure patients are dialysis-ready well in advance of actually starting a patient on dialysis. Likewise, starting prospective dialysis patients on an outpatient dialysis schedule (where costs are lower), or making use of urgent-start peritoneal dialysis programs [25], and avoiding traumatic scenarios, whereby dialysis patients require emergency care and consequently incur a minimum five-day hospital stay, could significantly lower total costs for patients with CKD.

Some efforts to reduce the costs associated with caring for patients with CKD are already underway. The Centers for Medicare and Medicaid Services (CMS) ESRD Seamless Care Organization is the first disease specific Accountable Care Organization designed by CMS to identify, test, and evaluate new ways to improve care for Medicare beneficiaries with ESRD. Likewise, the Large Dialysis Organizations are developing strategies to reduce costs incurred by CKD patients by identifying "high spenders". These include reaching out to such patients to remind them of their dialysis appointments and helping them with other disease related activities, such as managing their medications, diet, and transportation needs to and from appointments. Additionally, some dialysis providers are developing methods to mine their databases to identify “high spenders" and build economic models to determine what CKD care is best. Future research should examine the efficacy of these efforts and investigate how to best scale up successful aspects of these efforts.

We found that total costs for patients with CKD are significantly affected by co-morbid conditions (such as hypertension, diabetes, and other heart disease) and their associated costs. Cardiovasular disease and diabetes mellitus are the two leading causes of CKD. Better management of hypertension and diabetes mellitus, common co-morbidities associated with CKD, would slow the progression of kidney disease and reduce healthcare expenditures. Awareness of potential side effects due to the medications or renal insufficiency could prevent unnecessary harm to patients and provide cost-containment. Active involvement of all healthcare team members can reduce progression of CKD and improve quality of life outcomes in CKD patients. Moreover, while it is not surprising that more medically complex CKD patients incur higher costs, it reinforces the importance of delivering coordinated patient-centered care that is attentive to the whole person including the management of polypharmacy, the use of multiple medications and/or the administration of more medications than are clinically indicated. Polypharmacy continues to increase in the U.S. and is a known risk factor for morbidity and mortality. Additionally, treatment plans for CKD patients that activate and educate patients, family members and other care providers to better manage co-morbid conditions and adhere to targets established by physicians should be at the forefront of cost reduction strategies for treating individuals with CKD.

Our findings suggest that reducing the number of in-person visits and the number of prescription drugs might also reduce total costs per CKD patient. Reducing the frequency of in-person visits could be accomplished by using computer technologies, such as secure messaging (email) or telehealth systems, for non-essential office visits. Many medical systems are already using secure messaging systems to share the results from routine medical tests with patients [26, 27]. Reducing costs associated with prescription drugs is perhaps more difficult to address. One path to lowering the number of prescription medications used in the CKD population could come from better management of co-morbid conditions, as noted above. Another way could be through better efforts to more systematically address polypharmacy in this patient population. Predicting which CKD patients will end up on dialysis is very difficult. Often, patients at stage 3 or stage 4 will never receive dialysis. For every five to seven CKD patients seen, only one will end up on dialysis. The others will either not progress and/or die (usually of cardiovascular disease). Efforts might be best spent focusing on the cardiovascular health of the CKD patient population with less costly solutions (e.g. statins, blood pressure control, nutrition and exercise counseling) as a way to reduce long-term costs associated with co-morbid conditions.

Our findings also suggest that better care and reduced total costs for caring for patients with CKD could come from applying approaches to care that are grounded in systems thinking [28]. The majority of the frameworks applied to understand health care delivery systems emphasize the linear relationships among system components, where all variables and their relative weights are known [29]. Acknowledging the presence of nonlinear relationships and unpredictability in CKD treatment could help the individuals and organizations more accurately conceptualize the impact of various components of CKD care. Along these lines, models of care such as Patient-Centered Medical Homes, Accountable Care Organizations and Home-Based Primary Care are a step in the right direction for achieving a more integrated and holistic view of the complex needs shared by high-cost, high need patients with chronic disease. Such a reconceptualization of CKD processes and care trajectories could provide decision makers and policy makers with new insights into observed gaps in care and for visualizing novel paths forward for implementing CKD treatments and interventions more effectively.

### Limitations and future research

The above analyses and conclusions are relevant steps toward understanding and minimizing cost of care for CKD patients. However, there are enhancements not addressed in this paper that may be worthy of further research.

First, the models could be adapted to account for various co-morbid *combinations* (or interactions) and their subsequent impact on costs. For instance, high blood pressure and obesity are two variables that could jointly influence the type of treatment to which a patient is subjected, which in turn would have a differing impact on costs. Introducing interaction variables, however, would require a much larger dataset since one would have to include more independent variables. Second, it would be interesting to develop a time-series model for total costs that would allow one to project expenditures at least two or three years into the future. Kidney disease is progressive as are its attendant costs. Thus, patients would be better informed if at least some costs were predicted ahead of time. Such long-term prediction models, while useful, are non-trivial to develop since, when viewed as a time series, expenditure data are highly non-stationary. Third, assessing percentage changes to total costs by changing existing protocols is worthy of study. For instance, suppose the source-of-care provider days were increased by 5%. How would that increase impact the total cost to patients by different treatment types? This type of sensitivity analysis could help providers evaluate the tradeoffs between cost and quality of care decisions. Fourth, our results suggest that costs of treating CKD patients are lower for those with private as opposed to public insurance. Research assessing this relationship perhaps comparing costs pre and post the recent U.S. Affordable Care Act is needed. Fifth, in this paper we wanted to understand CKD costs before the roll-out of the ACA whose impact on CKD patient behavior and treatment started to alter in 2013. Given the dynamics of the current election cycle, this wait-and-see approach is better justified. Also, we wanted a clean, comprehensive data that reflected some important considerations such as: use of Erythropoietin stimulating agents (which was at its peak in 2006); growing momentum for payment policy reform in certain states; and moving toward bundled payments. Some of these criteria were corrupted in data starting roughly around 2012–2013. Nonetheless, it would be useful to revisit certain aspects of this research using new data. Sixth, it would be useful to investigate the CKD cost data across different countries, given the different health care plan choices available in different nations. Perhaps, one could adapt the U.S. model for CKD costs by borrowing strength from the analysis of such data from other countries: a meta-analysis study may be appropriate to address this research issue. Finally, insights from this study could be used to inform future research to understand and manage the cost-quality tradeoff for other complex and debilitating chronic conditions such as epilepsy, asthma, and heart disease.

## Conclusions

Managing CKD patients both before and after the onset of dialysis treatment and managing co-morbid conditions in individuals with CKD are potential sources of substantial cost savings in the care of CKD patients. Future research comparing total costs pre and post the U.S. Affordable Care Act could provide invaluable insights into managing the cost-quality tradeoff in CKD care.

## Abbreviations

AHRQ: Agency of Healthcare Research and Quality
BiVad: Biventricular heart assist devices
CKD: Chronic kidney disease
CMS: Centers for Medicare and Medicaid Services
ESRD: End-stage renal disease
ICD9CODX: International statistical classification of diseases version 9 code for condition
MEPS: Medicare expenditure panel survey
NDD-CKD: Non-dialysis dependent chronic kidney disease
pmp: Per million populations
